# The impact of oral isotretinoin on ovarian functions of acne patients complaining of polycystic ovarian syndrome: a prospective study

**DOI:** 10.1186/s13048-024-01347-x

**Published:** 2024-01-20

**Authors:** Heba I. Elnagar, Osama A. Hashem, Hany O. Aboelwafa, Ehab Elhelw, Mohamed L. Elsaie

**Affiliations:** 1 Department of Dermatology, Venereology and Andrology, Damietta Faculty of Medicine, Al-Azhar University, Damietta, Egypt; 2 Department of Obstetrics and Gynecology, Damietta Faculty of Medicine, Al-Azhar University, Damietta, Egypt; 3 Department of Dermatology, Medical Research and Clinical Studies Institute, National Research Centre, Cairo, Egypt

**Keywords:** Isotretinoin, Acne, PCOS

## Abstract

**Background:**

Women have polycystic ovarian syndrome (PCOS) at higher rates than any other endocrine condition with an average incidence rate of 6 to 8%. Acne is an immune mediate common condition frequently affecting adolescents and adults and is often associated with PCOS. The objective of the study was to assess the impact of oral isotretinoin on ovarian functions of acne patients suffering from PCOS. Forty women with a clinical diagnosis of acne as well as PCOS participated in this prospective clinical trial. Participants were given oral doses of isotretinoin ranging from 0.5 to 1 milligram per kilogram (mg/kg), for a total of 120 to 150 mg/kg. To establish baseline values of hormone levels, on days 2–5 of the menstrual cycle, venous blood samples were obtained. Moreover, global acne grading system (GAGS), follicle count, and bilateral ovarian volumes were evaluated both before and after isotretinoin treatment.

**Results:**

A significant reduction in global acne score from pre-treatment levels to post-treatment levels was observed (11.58 ± 5.857 vs. 1.65 ± 1.369). Ovarian volume was significantly reduced from 10.26 ± 1.539 before treatment to 8.74 ± 1.436 after treatment on the right side (*P* < 0.001) and from 11.08 ± 1.564 before treatment to 9.36 ± 1.479 after treatment on the left side (*P* < 0.001). A significant reduction in free testosterone level and hirsutism scores were observed after treatment (*P* < 0.001; *P* < 0.01 respectively.

**Conclusion:**

Isotretinoin may exert beneficial effects in hyperandrogenic women with PCOS and needs to be further evaluated by large multicentre controlled trials.

## Background

Acne is an immune mediate common condition frequently affecting adolescents and adults. It predominately affects the head, neck and body. Severe acne can be challenging to treat and oral isotretinoin is one modality available for cure or sustained remission in moderate and severe cases [1]. Isotretinoin decreases sebaceous gland secretion, induces apoptosis of bacterial cells and sebocytes and further normalize the desquamation of epithelial cells and therefore prevent comedone formation. Moreover isotretinoin exhibits antinflammatory properties [2]. Isotretinoin had been shown to promote its apoptotic effects via the increased expression of transcription factors p53, FoxO1 and FoxO3 [3].

Women have (PCOS) at higher rates than any other endocrine condition with an average incidence rate of 6 to 8%. As a common symptom, acne vulgaris (AV) can affect up to 62% of adolescents with PCOS [4]. PCOS is routinely assessed using the Rotterdam criteria; yet precise diagnostic criteria remains to be questionable by many authors [5–7]. According to the Rotterdam criteria, the diagnosis of PCOS may present in patients with four different phenotypes: (1) hyperandrogenism, chronic anovulation, and polycystic ovaries; (2) hyperandrogenism and chronic anovulation but normal ovaries; (3) hyperandrogenism and polycystic ovaries but ovulatory cycles; and (4) chronic anovulation and polycystic ovaries but no clinical or biochemical hyperandrogenism [5].

Isotretinoin has been shown to reduce insulin growth factor (IGF-1) that induces the expression of 5-α reductase and converts testosterone to dihydrotestosterone (DHT). This reduction in androgen activity is explained by isotretinoin mediated upregulation of P53 expression [8]. Thus, it should come as no surprise that isotretinoin causes a decrease in testosterone levels in acne patients taking the medication. Metformin; used in treating PCOS similarly increases p53 expression in PCO patients and based on this assumption, isotretinoin can similarly be of value in PCOS [9].

Recent studies have shown that isotretinoin enhances granulosa cell apoptosis reducing follicular reserve. Since isotretinon inhibits p53 & has an antiproliferative effect on ovarian stromal cells, its use can lead to a reduction in ovarian volume. Furthermore; experimental evidence indicates that isotretinoin might suppress the pituitary-ovarian axis at the level of gonadotropin expression as well as granulosa cell homeostasis [10].

The focus of this research was to better understand the effectiveness of isotretinoin for the treatment of PCOS symptoms.This study’s objective was to determine effectiveness of oral isotretinoin in treating acne and PCOS in female subjects.

## Methods

Clinics for both dermatology and gynecology at Al-Azhar University (Damietta) were used for this prospective clinical investigation. Forty women above the age of sixteen who suffered from acne and polycystic ovarian syndrome were included.

Clinical diagnosis of acne vulgaris was performed using the “Acne vulgaris: review and guidelines 2009” [11]. Acne severity was evaluated in patients group using Global Acne grading System (GAGS). Participants with GAGS scores of 18 or less were classified as having mild acne, those with GAGS scores of 19 to 30 as having moderate acne and individuals with GAGS values of 31 or more as having severe acne [12]. PCOS was defined according to the Rotterdam criteria [5]. Hirsutism was assessed using the modified FerrimanGallwey (F–G) score, in which a score > 8 indicates hirsutism. Hirsutism is the most consistent and reliable symptom used for evaluating clinical hyperandrogenism. Ferriman and Gallwey described a visual scoring method to clinically assess the degree of hirsutism known as the Ferriman-Gallwey (FG) score. According to the FG score, hair is scored in nine parts of the body, which include the upper lip, chin, chest, upper and lower back, upper and lower abdomen, and upper and lower limbs. A score of 0–4 is given on these nine body parts to determine the extent of hirsutism, with a score of 0 representing a complete absence of terminal hair and a score of 4 represents extensive hair growth. The score of all nine areas is added up to get the final score used for diagnosis. Women with an FG score of 8 or higher are regarded as hirsute [13]. Hyperandrogenism was defined as a modified hirsutism score of ≥ 7 and/or an elevated total testosterone value of ≥ 3.96 nmol/L. Exclusion criteria included the following: antidepressant usage, Steroid hormone medications, mood stabilizers, alcohol, or tobacco; previous ovarian surgery or abdominal surgery for endometriosis; existence of any systemic illness contraindicated with isotretinoin therapy (e.g., hyperlipidemia); presence of infectious diseases (e.g., TB, HCV); and use of isotretinoin.

Following the study’s approval by the local ethics committee at the Faculty of Medicine at Al-Azhar University in Damietta, all cases included in the study provided their verbal and written informed consent. A detailed medical history was taken, a physical examination was performed and a dermatological examination carried out on every individual being examined.

### Baseline assessment

During the menstrual cycle’s days 2–5, blood was drawn in the morning following a 6-hour fast to measure hormone levels and other biochemical parameters.

Each participant had venous blood drawn for testing. After drawing blood, we centrifuged the samples at 3000 rpm for 5 min to separate the serum. Before the research day, serum samples were put in Eppendorf tubes and frozen at -20 °C in the lab’s deep freezer.Total testosterone (TT), free testosterone (FT), and luteinizing hormone (LH) levels, as well as thyroid stimulating hormone (TSH), follicle stimulating hormone (FSH), luteinizing hormone (LH), estradiol (E2) and dehydroepiandrosterone sulfate (DHEAS). An automated chemiluminescence system was used to determine the levels of AST, ALT, cholesterol and triglycerides in the serum. From Day 3 and Day 7 of the menstrual cycle, ultrasounds were carried out. All scans were done in a private room after obtaining the patient’s consent using a Voluson E6 (GE Healthcare, Zipf, Austria) diagnostic ultrasound system using a 2–5 MHz abdominal probe. Each ovary was seen and its position relative to the utero-ovarian ligament was determined anatomically. Both the transverse and sagittal planes were utilized to examine the ovaries from their innermost to most outermost edges. The size of the ovaries, the number of follicles present, the diameter of the largest follicle, and the pattern of follicular distribution was measured and analyzed.

### Treatment regimen and follow up

Participants were given oral doses of isotretinoin ranging from 0.5 to 1 milligram per kilogram (mg/kg), for a total of 120 to 150 mg/kg. Ultrasonography was used to check up on the individuals at baseline and after three and six months of treatment respectively.

### Statistical analysis of data

Statistical Package for Social Sciences (SPSS) version 27 for Windows® was used to code, process, and analyze the information that was obtained (SPSS Inc, Chicago, IBM, IL, USA). Quantitative information was presented as a number (frequency) and a percentage. The Chi-Square test (also known as the Fisher’s exact or Monte-Carlo test) was used tfor comparison of the groups. Quantitative data were examined for normality using the Kolmogorov-Smirnov test.While parametric data were shown as a mean ± SD, non-parametric data were shown as a median (interquartile range). P values below 0.05 are deemed significant.

## Results

This research was a prospective clinical investigation involving forty women clinically diagnosed with acne as well as polycystic ovarian syndrome. Individuals were recruited from Al-Azhar University’s dermatology and gynecology outpatient clinic in Damietta. Age of included participants ranged from (16–27 years old) with mean age of 21.18 ± 3.289. BMI ranged from (24.37–33.97 kg/m^2^) with a mean of 29.31 ± 3.005.

Twenty seven women (67.5%) had hyperandrogenism, 30 (75%) of them had hirsutism, 28 (70%) of them had unusual testosterone level, 26 (65%) had oligomenorrhea or amenorrhea and 31 (77.5%) had PCO confirmed by ultrasonography. The dose of isotretinoin prescribed varied from (20 to 60 mg/kg) and the mean was (38.0 ± 14.178) mg/kg. There was a statistically significant decrease in acne score following treatment (2.42 ± 0.844 vs. 0.73 ± 0.452, *P* < 0.001). Also there was a significant decrease global acne score after treatment (11.58 ± 5.857 vs. 1.65 ± 1.369, *P* < 0.001). Tables 1 and 2.

**Table 1:** Signs of PCOS of the investigated sample

All patients (*n*= 40)		Frequency	Percentage
Hyperandrogenism		27	67.5
Hirsutism		30	75.0
Abnormal Testosterone level		28	70.0
Oligomenorrhea or amenorrhea		26	65.0
Polycystic ovary by US		31	77.5
Rotterdam phenotypes	Type I classic PCOS (A, HA, PCO)	4	10.0
	Type II classic PCOS (A, HA)	9	22.5
	Ovulatory PCOS (HA, PCO)	14	35.0
	Normoandrogenic PCOS (A, PCO)	13	32.5

Data is expressed as percentage and frequency

**Table 2:** Comparison of baseline acne score acne global score and laboratory investigations at baseline and after treatment

	Baseline (*n*= 40)	After treatment (*n*= 40)	95% CI	P
M F-G Score	11.02 ± 3.02	10.16 ± 2.32	0.19. 0.17	*<0.01
Acne score	2.42 ± 0.844	0.73 ± 0.452	1.4, 2.0	*<0.001
The global score	11.58 ± 5.857	1.65 ± 1.369	8.0, 11.8	*<0.001
FSH	7.00 ± 0.558	7.00 ± 0.593	-0.07, 0.07	0.959
LH	5.43 ± 1.126	5.36 ± 1.127	-0.06, 0.18	0.294
E2	48.43 ± 8.755	48.72 ± 9.045	-0.80, 0.23	0.270
DHEAS	216.35 ± 29.834	214.17 ± 31.406	-0.08, 4.45	0.058
TT	0.47 ± 0.062	0.47 ± 0.061	0.00, 0.01	0.377
FT	1.82 ± 0.286	1.56 ± 0.253	0.21, 0.30	*<0.001
AST	23.72 ± 3.093	23.77 ± 3.287	-0.30, 0.20	0.678
ALT	22.40 ± 1.930	22.33 ± 1.984	-0.16, 0.29	0.552
Cholesterol	138.08 ± 15.029	160.32 ± 20.002	-25.08, -19.39	*<0.001
TG	119.38 ± 7.500	138.79 ± 11.474	-21.89, -16.93	*<0.001

MF-G Modified Ferriman-Gallwey Score; FSH: follicle stimulating hormone, LH: luteinizing hormone, E2: estradiol, DHEAS: dehydroepiandrosterone sulfate, TT: testosterone, FT: free testosterone, AST: alanine transaminase, AST: aspartate transaminase, TG: triglycerides, CI confidence level of 95%, p: probability, *statistically significant if *p* < 0.05

A significant reduction in free testosterone level and hirsutism scores were observed after treatment (*P* < 0.001; *P* < 0.01 respectively); while a significant increase in serum levels of both triglycerides and cholesterol was demonstrated (*P* < 0.001). Ovarian volume was significantly reduced from 10.26 ± 1.539 before treatment to 8.74 ± 1.436 after treatment on the right side (*P* < 0.001) and from 11.08 ± 1.564 before treatment to 9.36 ± 1.479 after treatment on the left side (*P* < 0.001). Similarly right follicular count decreased from 10.28 ± 1.198 before treatment to 8.05 ± 1.339 after treatment (*P* < 0.001) while left follicular count decreased significantly from 9.55 ± 1.584 before treatment to 7.68 ± 1.457 after treatment (*P* < 0.001). Table 3.

**Table 3:** Comparison between baseline and after treatment ovarian volume (OV) and antral follicular count (AFC)

	Baseline (*n*= 40)	After treatment (*n*= 40)	95% CI	P
Right ovarian volume	10.26 ± 1.539	8.74 ± 1.436	1.27, 1.76	*<0.001
Left ovarian volume	11.08 ± 1.564	9.36 ± 1.479	1.49, 1.96	*<0.001
Right follicular count	10.28 ± 1.198	8.05 ± 1.339	1.90, 2.55	*<0.001
Left follicular count	9.55 ± 1.584	7.68 ± 1.457	1.53, 2.22	*<0.001

OV: ovarian volume, AFC: antral follicular count, CI confidence level of 95%, p: probability, *statistically significant if *p* < 0.05

## Discussion

Acne scores were significantly varied before and after therapy in the current research (2.42 ± 0.844 vs. 0.73 ± 0.452, *P* < 0.001). Additionally, there was a statistically significant difference in the global acne score (11.58 ± 5.857 vs. 1.65 ± 1.369, *P* < 0.001). This was consistent with Acmaz et al. who looked into how isotretinoin affected PCOS patients with severe cystic acne and reported a significant decrease of the mean acne after treatment (*P* < 0.01) [14].

Both AST and ALT levels were comparable before and after therapy in the current study (23.72 ± 3.093 vs. 23.77 ± 3.287, *P* = 0.678 and *P* = 0.552, respectively). This comes in contrast to the findings of Vieira et al., who reported a significant increase in AST (20.44 ± 6.26 vs. 24.38 ± 11.92 U/L) and ALT (18.24 ± 8.31 vs. 23.34 ± 20.03 U/L) after using isotretinoin for 3 months [15]. A significant increase in serum levels of both triglycerides (119.38 ± 7.500 vs. 138.79 ± 11.474, *P* < 0.001) and cholesterol (138.08 ± 15.029 vs. 160.32 ± 20.002, *P* < 0.001) was demonstrated in the present study. This agrees with the findings of Acmaz et al. [14], Ahmadvand et al. [16], and Brito et al. [17] who noticed that triglyceride and cholesterol significantly increased after treatment with isotretinoin and with Vieira et al. [15] who reported significant serum triglyceride increase after three month of isotretinoin therapy.

The precise cause of isotretinoin-induced lipid elevation is unknown and remains to be elucidated; however it is believed that retinoids such as isotretinoin can displace triglycerides from plasma albumin as well as their potential to affect enzymes involved in lipid metabolism [18]. Given the fact that a significant increase in TG and cholesterol could be encountered; isotretinoin treatment may be beneficial in patients with PCOS and acne who are not suffering of any lipid profile disturbances and requires regular follow ups.

The current study showed that there were non-significant differences between baseline and after treatment for LH, FSH, E2 as well as DHEAS while a significant reduction in free testosterone after treatment was reported (*P* < 0.01). This was in contrast to Öztürk and colleagues who evaluated ovarian reserve in women with severe acne taking oral isotretinoin and noticed that the mean FSH, LH, and E2 levels were significantly lower than before treatment [19]. It is worth mentioning that they used a higher dose regimen of 0.5-2 mg/kg.

An earlier study aimed on verifying the benefits of isotretinoin for treating acne in hyperandrogentic women complaining of PCOS. Eight (8) of ten (10) women complaining of nodulocystic acne and diagnosed with PCOS completed a 20 weeks course of isotretinoin and showed a significant reduction in their acne and testosterone levels [20].

A prospective study investigated isotretinoin treatment (0.5 − 0.75 mg/kg/day) for three months in acne patients (16 males; 31 women) and evaluated the pituitary-adrenal axis after isotretinoin treatment. A significant reduction of LH, PRL, and total testosterone was reported. This was associated with the thyroid and adrenal dysfunction [21]. A recent study group demonstrated significantly lower levels of FSH, LH and E2 in 32 female patients complaining of severe acne and treated by 0.5 -2 mg/kg /day of oral isotretinoin [22].

The current research reported a significant decrease in ovarian volume (OV) and antral follicular count (AFC) after treatment. In women with PCOS, multiple small follicles (small cysts 4 to 9 mm in diameter) accumulate in the ovary. None of these small follicles/cysts are capable of growing to a size that would trigger ovulation. As a result, the levels of estrogen, progesterone, LH, and FSH become imbalanced [20]. Such findings came in accordance with findings of Acmaz et al. who observed significant decrease in right and left ovarian volume and antral follicular count [14]. Haroun et al. assessed the effect of low dose isotretinoin on the ovarian reserve in 66 female patients with moderate to severe acne. Both OV and AFC showed no significant changes in patient group when comparing pre- post- treatment levels on both sides (*p* > 0.05). They concluded that low-dose - isotretinoin in treatment of moderate to severe acne seems to be safer on ovarian reserve [23]. The difference in results could be due to the lower dose used ( 0.25-0.4 mg/Kg) used and needs to be verified by further studies.

In their study to investigate the effects of isotretinoin treatment in women with acne on ovarian function based on OV and AFC, Öztürk et al. reported that while the mean number of antral follicles and ovarian volume both dropped following therapy, the differences were not statistically significant [19]. Another study performed on 79 patients reported a non significant LH, and FSH variance but demonstrated a significant reduction in OV and AFC. Of note, these reductions were reversible 12 months after termination of isotretinoin administration [24]. Once again; this controversy may be due to different dosage regimens.

Isotretinoin was recently linked with reports assuming that it affects pituitary ovarian access and can modify pituitary hormone levels [24]. Rat studies demonstrated isotretinoin to reduce ovarian reserve. Moreover, the retinoid signaling pathway has been linked to various female reproductive pathologies including endometriosis and polycystic ovary syndrome (PCOS) [25].

The current study is limited by its small sample size and the lack of long-term patient follow-up. Moreover; it had been a single arm uncontrolled study.

## Conclusion

In conclusion this current study highlights an important aspect of isotretinoin’s potential impact on the pituitary ovarian axis. Isotretinoin may exert beneficial effects in hyperandrogenic women with PCOS and needs to be further evaluated on large multicentre controlled trials. Considering the difficulties and delay in conception by women complaining of PCO’s, it is of importance, to consider short and long-term effects of isotretinoin treatment on fertility in adolescent and adult women and consider its teratogenic effects as a possible limitation to its use.

## Data Availability

The data that support the findings of this study are available from the corresponding author upon reasonable request.

## Abbreviations

PCOs: Polycystic ovarian syndrome
FSH: Follicle stimulating hormone
LH: Luteinizing hormone
E2: Estradiol
DHEAS: Dehydroepiandrosterone sulfate
TT: Testosterone
FT: Free testosterone
AST: Alanine transaminase
AST: Aspartate transaminase
TG: Triglycerides
OV: Ovarian volume
AFC: Antral follicular count
GAGS: Acne grading System
